# Identifying and modulating distinct tremor states through peripheral nerve stimulation in Parkinsonian rest tremor

**DOI:** 10.1186/s12984-021-00973-6

**Published:** 2021-12-25

**Authors:** Beatriz S. Arruda, Carolina Reis, James J. Sermon, Alek Pogosyan, Peter Brown, Hayriye Cagnan

**Affiliations:** Medical Research Council Brain Network Dynamics Unit, Nuffield Department of Clinical Neurosciences, University of Oxford, Mansfield Road, Oxford OX1 3TH, UK

**Keywords:** Parkinson’s disease, Peripheral stimulation, Non-invasive, Phase-locked stimulation, Tremor oscillation patterns

## Abstract

**Background:**

Resting tremor is one of the most common symptoms of Parkinson’s disease. Despite its high prevalence, resting tremor may not be as effectively treated with dopaminergic medication as other symptoms, and surgical treatments such as deep brain stimulation, which are effective in reducing tremor, have limited availability. Therefore, there is a clinical need for non-invasive interventions in order to provide tremor relief to a larger number of people with Parkinson’s disease. Here, we explore whether peripheral nerve stimulation can modulate resting tremor, and under what circumstances this might lead to tremor suppression.

**Methods:**

We studied 10 people with Parkinson’s disease and rest tremor, to whom we delivered brief electrical pulses non-invasively to the median nerve of the most tremulous hand. Stimulation was phase-locked to limb acceleration in the axis with the biggest tremor-related excursion.

**Results:**

We demonstrated that rest tremor in the hand could change from one pattern of oscillation to another in space. Median nerve stimulation was able to significantly reduce (− 36%) and amplify (117%) tremor when delivered at a certain phase. When the peripheral manifestation of tremor spontaneously changed, stimulation timing-dependent change in tremor severity could also alter during phase-locked peripheral nerve stimulation.

**Conclusions:**

These results highlight that phase-locked peripheral nerve stimulation has the potential to reduce tremor. However, there can be multiple independent tremor oscillation patterns even within the same limb. Parameters of peripheral stimulation such as stimulation phase may need to be adjusted continuously in order to sustain systematic suppression of tremor amplitude.

## Background

Approximately 70% of the people with Parkinson’s disease exhibit involuntary shaking of their limbs when resting [1]. Involuntary shaking of the limbs, also known as tremor, can dominate Parkinson’s disease, and yet responds less well to dopaminergic medications than bradykinesia and rigidity [2, 3]. Deep brain stimulation can provide striking tremor relief however this surgical intervention is invasive and subject to strict selection criteria, which can limit the number of people benefiting to approximately 2% [4, 5]. As a result, there has been growing interest in non-invasive therapies for tremor in Parkinson’s disease. Therapies directly interfacing with the limb rather than cranial stimulation [6, 7] are more tractable and have focused on occluding tremor by stimulation of antagonist muscles or suppressing tremor by stimulation of sensory afferents. However, these approaches can lead to incomplete and unpredictable tremor suppression, muscle fatigue and discomfort, and have not entered in to clinical practice [8–13]. Vibratory stimulation is another non-invasive approach, which has been shown to improve motor performance in people with Parkinson’s disease; however, its impact specifically on rest tremor has not been quantified extensively [14].

It has recently been demonstrated that brain rhythms can be modulated using phase-locked stimulation [15–19]. This stimulation strategy aims to selectively enhance or reduce a rhythm depending on the precise stimulation timing with respect to the target rhythm. In the case of tremor, phase-locked stimulation has been trialled in the form of cranial or deep brain stimulation locked to peripheral tremor, which significantly modulated tremor severity [4, 6, 7, 20]. Previous research suggests that delivering stimulation to the median nerve can cause neural spiking in the thalamic nuclei, which overlap with regions implicated in tremor in Parkinson’s disease and are commonly used as surgical targets for deep brain stimulation [21–25]. Critically, thalamic neurons fire at a certain instance of limb acceleration during tremor which raise the possibility that median nerve stimulation phase-locked to limb acceleration could potentially impact the central oscillators underpinning Parkinsonian tremor [23, 26].

An important consideration for peripheral nerve stimulation is the relationship between central tremor oscillators and the peripheral manifestation of tremor. Currently, it is believed that resting tremor in Parkinson’s disease is generated by independent central oscillators separately representing each limb. Several studies of multi-limb tremor recordings indicate that tremor in different limbs is largely uncorrelated, while being correlated within a limb [27–31]. It has recently been shown that intermuscular coherence can be used to distinguish between different tremor types and correlates with tremor intensity in Parkinson’s disease [32, 33]. In a small sample of people with Parkinson’s disease, the mean coherence between the axes of an accelerometer placed on a tremulous limb was 0.72 and the coherence between signals obtained from accelerometers placed on different regions of the same limb was only 0.56 [28]. There are two possible explanations for these observations: (1) coupling within a limb-specific central tremor oscillator drops in and out over time, or (2) multiple central tremor oscillators contribute to rest tremor even within the same limb, perhaps with one or more dominating at any particular time. Studies of the coherence between microelectrode recordings within the globus pallidus or subthalamic nucleus (STN) and electromyography (EMG) in people with Parkinson’s disease provide evidence for the former [34, 35]. A recent modelling study has also highlighted that one central tremor drive is sufficient to capture features derived from experimental recordings [36]. On the other hand, the presence of spatially distinct pockets of coherence with tremor within the STN and ventral thalamus raises the possibility of multiple competing central oscillators [37, 38]. The nature of the central representation of tremor is important for the insights it may afford into motor control and the development of novel therapeutic interventions such as phase-locked stimulation [15, 16, 39, 40]. Afferent feedback is also critical in tremor pathophysiology. As the basal ganglia circuits involved in tremor are topographically arranged it is likely that the effects of afferent feedback will also be topographically limited [41–45].

Here we test two major hypotheses which are interlinked; that the amplitude of Parkinson’s disease rest tremor in the hand can be modulated by phase-locked stimulation of a peripheral nerve supplying the hand, and that tremor in the hand can involve multiple oscillators. To this end, we electrically stimulated the median nerve at the wrist in people with Parkinsonian rest tremor to show that tremor amplitude modulation can be achieved with phase-locked stimulation, provided that the existence of multiple oscillators is taken into account.

## Methods

### Cohort

We recruited 14 people with Parkinson’s disease who displayed upper-limb tremor at rest. All participants were recruited by an advert placed with an advocacy group for people with Parkinson’s disease. Study participants were assessed by an experienced movement disorders expert at the time of study (PB). This led to the exclusion of one person in whom the diagnosis of dystonia and one in whom the diagnosis of psychogenic tremor was made. Two further participants were excluded; one due to minimal tremor confined to the little finger, and the other as a result of tremor transmitted from the legs. Thus, we analysed data from 10 participants (Hoehn and Yahr Scale stages I and II). Eight out of 10 of the participants regularly took medication for Parkinson’s disease and five of these eight participants omitted their last dose prior to the experiment. The three participants who chose not to omit their medication dose reported that their upperlimb tremor was unaffected by medication and exhibited upper-limb tremor at rest at the time of the study. Table 1 summarises participant information.

### Data acquisition

**Fig. 1:**
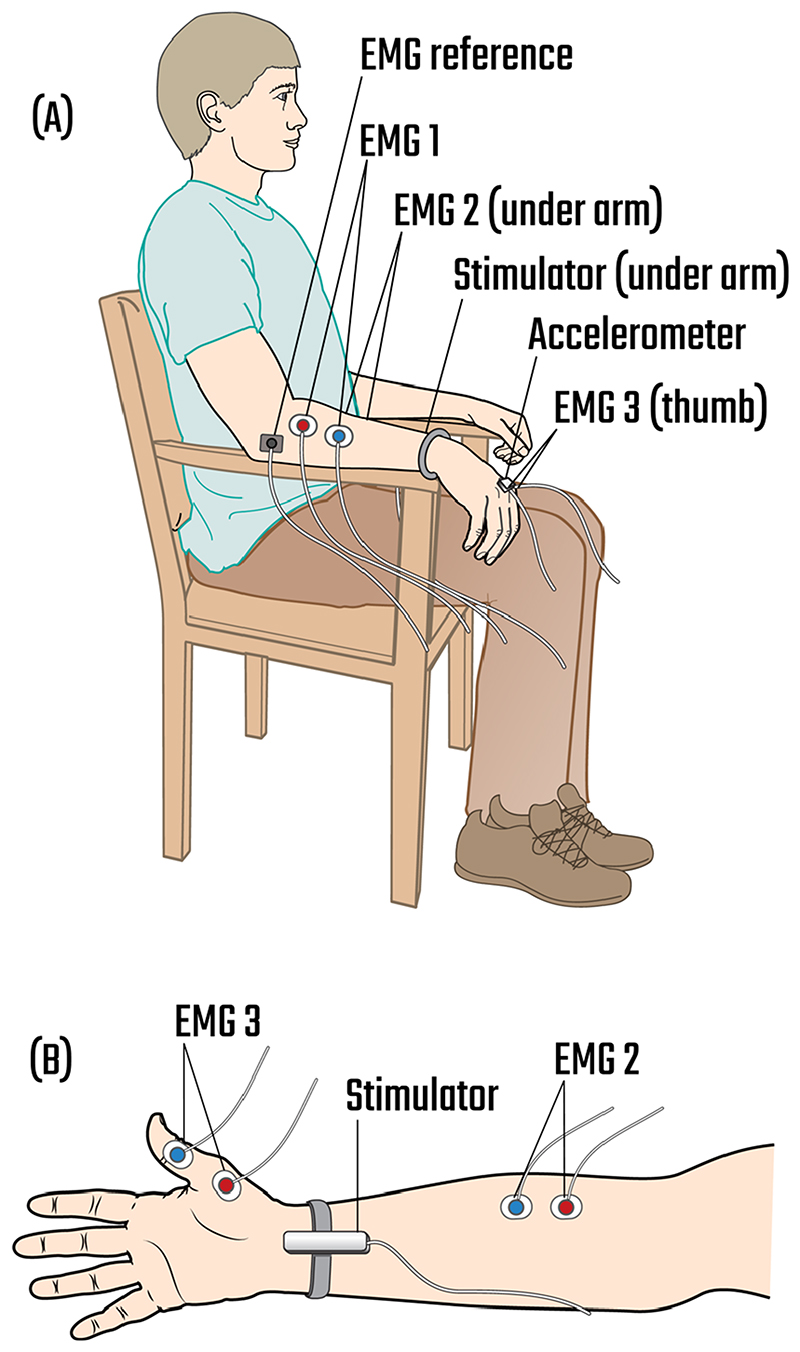
An illustration of the triaxial accelerometer, peripheral stimulation electrodes, and EMG surface electrodes placed on participant’s upper limb. **A** Participant’s hand was hanging over the armrest of the chair which supported the forearm. A triaxial accelerometer was placed on the dorsum of the most tremulous hand, a wristband containing stimulation electrodes was attached to the wrist, and EMG surface electrodes were placed on the participant’s hand and forearm. EMG 1 recorded signals from the forearm finger extensors; EMG 2, from the forearm finger flexors; and EMG 3, from the abductor pollicis brevis. **B** An illustration of the ventral surface of the study participant’s forearm and hand, showing the placement of the stimulation electrodes and EMG 2 and 3 surface electrodes

Resting tremor was recorded in two conditions (1) without stimulation and (2) during peripheral nerve stimulation, while study participants were sitting. Participants were asked to avoid voluntary movements during the recording. A triaxial accelerometer (ACL300 Biometrics Ltd) was fixed to the dorsum of the most tremulous hand using surgical tape and tremor was recorded with the hand hanging over the side of the chair which supported the forearm. The forearm was placed on the armrest of the chair such that it was supported up to the wrist (Fig. 1A). In this position, the tremulous hand was hanging over the front of the armrest and could move without obstruction. The accelerometer signal was amplified (K800 Biometrics Ltd) and recorded using an analogue-to-digital (AD) converter (Power1401, Cambridge Electronics Design) at a sampling rate of 10,417 Hz. This sampling rate was chosen for phase estimation which relied on zero-crossings of the band-pass filtered accelerometer signal (see section ‘Peripheral nerve stimulation’) [39]. A wristband containing peripheral stimulation electrodes was also attached to the study participant’s wrist (Fig. 1B). EMGs were recorded from the abductor pollicis brevis, forearm finger flexors, and forearm finger extensors of the most tremulous arm using surface electrodes and an amplifier (D360 8 Channel Patient Amplifier, Digitimer) connected to an AD converter (Power1401, Cambridge Electronics Design). Seven surface electrodes were attached to the participant’s arm for EMG recordings (one pair for each muscle, plus the ground electrode), each electrode weighing 1 g. The accelerometer, attached to the dorsum of the most tremulous hand, weighed 10 g and the stimulation electrodes, attached to the wrist, weighed 16 g. Figure 1 shows the placement of the accelerometer, peripheral stimulation electrodes, and EMG surface electrodes on the participant’s upper limb. EMGs from one participant were not included in the analysis due to recording quality (assessed visually).

### Without stimulation condition

The recordings made in the without stimulation condition lasted on average 5.7 ± 1.8 min (mean ± SD). Each participant’s tremor frequency was identified as the frequency at which the power spectral density was the largest (2–8 Hz), using power spectral densities derived from the three accelerometer axes (Spike2, Cambridge Electronic Design Limited). The dominant axis was defined as the one with the largest power spectral density at the tremor frequency.

### Peripheral nerve stimulation

Stimulation amplitude was set to just below each participant’s motor threshold. Subject-specific motor threshold was determined by increasing the stimulation amplitude in steps of 0.5 mA from 2 mA until stimulation evoked a motor response (i.e., twitch of the thumb). The motor threshold ranged from 4 to 14 mA (mean 6.8 ± SD 1.8 mA) across our cohort. The twitch of the thumb was used to verify that the stimulation electrode was properly placed over the median nerve, which innervates the abductor pollicis brevis.

**Fig. 2:**
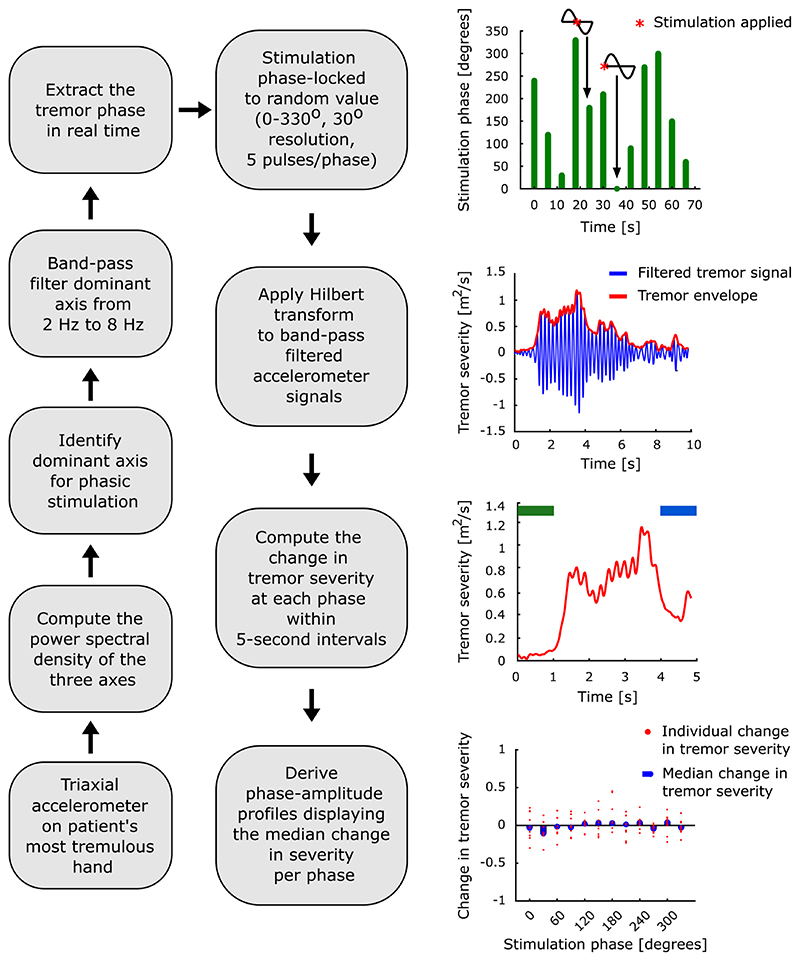
Data collection and analysis pipeline. Tremor signals were collected from the most tremulous hand using a triaxial accelerometer. The power spectral density of the three axes was computed, the dominant tremor axis was identified as the one with the largest peak at the frequency of the tremor and was subsequently band-pass filtered between 2 and 8 Hz. The tremor phase was extracted from the filtered signal in real time. Stimulation was phase-locked to one of 12 randomly assigned phases from 0 to 330° with a 30° resolution. To evaluate the effect of phase-locked peripheral nerve stimulation, Hilbert transform was applied to band-pass filtered accelerometer signals, from which change in tremor severity within 5-s epochs was computed by subtracting the average of the tremor envelope during the first 1 s of stimulation (indicated in green) from the average envelope during the last 1 s (indicated in blue) and then dividing the result by the average of the first 1 s (indicated in green). The phase-amplitude profiles were derived as the medians for all the changes at a given phase.

The dominant tremor axis, identified in the without stimulation condition, was band-pass filtered from 2 to 8 Hz (Digitimer NL125/6), and recorded with the AD converter (Power1401, Cambridge Electronics Design) at a sampling rate of 10,417 Hz for on-line phase-estimation. Stimulation phase was derived from the instantaneous zero crossings of this band-pass filtered signal and the average tremor frequency from the without stimulation condition. Once a desired stimulation phase was detected, a pulse was sent to the peripheral stimulator (Digitimer Constant Current Stimulator DS74), closing the loop. Stimulation at a certain phase consisted of a burst of five pulses spaced at 7.7 ms. Stimulation at each phase was presented for 5 s with a 1 s interval. Stimulation was locked to one of 12 equally spaced phases from 0 to 330° (Fig. 2). Stimulation phase order was randomised between different blocks. Stimulation blocks, consisting of stimulation at 12 different phases, were repeated 11–16 times with 1 min rest in between. Stimulation sensation was assessed using the following Likert scale: “The stimulation was easily tolerated: (A) Strongly disagree; (B) Disagree; (C) Neither agree nor disagree; (D) Agree; (E) Strongly agree”. Study participants’ responses are indicated in Table 1.

### Data processing and analysis

Recordings were analysed using custom written scripts in MATLAB (The MathWorks, Inc.). Signals corresponding to the three accelerometer axes were down-sampled to 1000 Hz. The tremor frequency was derived from Welch’s power spectral density using a window length of 1 s with no overlap for each condition (without stimulation and during peripheral nerve stimulation). Signals corresponding to each of the three accelerometer axes were band-pass filtered within ± 2 Hz of the peak tremor frequency using a second order Butterworth zero-phase digital filter (Fig. 2). EMG signals were down-sampled to 1000 Hz and then high-pass filtered at 15 Hz using a second order Butterworth zero-phase digital filter. We then rectified each filtered EMG signal. Following rectification, we only considered the EMG signal components greater than 1 Hz. Only EMG signals from the without stimulation condition were used for analysis as EMGs obtained during peripheral nerve stimulation were contaminated by stimulation artefacts.

### Tremor oscillation patterns

We sought evidence for discrete tremor oscillation patterns (TOPs). We define TOPs as different peripheral manifestations of tremor derived from the three accelerometer axes. To this end, band-pass filtered signals from the *x*, *y* and *z* accelerometer axes were subjected to principal component analysis (PCA). This procedure was used to extract axis-specific coefficients (i.e., loadings) contributing to the first principal component. PCA was applied to signals from both conditions (i.e., without stimulation and during peripheral nerve stimulation) after signals from each were divided into 5-s segments (Additional file 1).

In order to determine whether there was more than one TOP across the recording, we subjected the first principal component coefficients (loadings) to cluster analysis. The coefficients (3 by 1), indicating the contribution of three accelerometer axis to the first principal component of each 5-s recording segment, were concatenated across the two recording conditions (3 by *n*, where *n* is the total number of 5-s segments derived from both without stimulation and during peripheral nerve stimulation conditions). Cluster analysis was based on Euclidean distances and inner squared distances, seeking two clusters. We eliminated any clusters which contained less than 10% of the total number of segments from the peripheral nerve stimulation condition. Cluster silhouette values, which described how similar a point was to others in the same cluster with respect to points in other clusters, were used to characterise cluster separation. Silhouette values ranged from − 1 to 1 and high scores indicated that there was good cluster separation as the objects were better matched to their own cluster than to neighbouring clusters [46].

For the without stimulation condition, we computed the variance explained by each of the three principal components. In the cases in which the first principal component accounted for less than 90% of the variance (median across the 5-s epochs), we also subjected the second principal component coefficients (loadings) to the same cluster analysis. In these instances, we detected up to four TOPs per recording (two from the first principal component coefficients and two from the second principal component coefficients).

To evaluate whether there were any differences between TOPs, we computed the median tremor envelope and frequency across 5-s segments of each cluster for each study participant. The tremor envelope was defined as the absolute value of the Hilbert transform, and the tremor frequency was derived from the instantaneous unwrapped phase of the Hilbert transform. Cluster division was based on the principal component coefficients derived from the triaxial accelerometer signals recorded during the two experimental conditions (3 by *n*, where *n* is the total number of 5-s segments derived from both without stimulation and during peripheral nerve stimulation conditions). EMG epochs were assigned to clusters, defined according to the loadings of the simultaneously recorded accelerometer signals. Only data from the without stimulation condition was considered for statistical comparison since it was not feasible to analyse EMGs recorded during stimulation. It should be noted that clusters which contained less than 10% of the total number of segments from the peripheral nerve stimulation condition were retained for this analysis since differences between TOPs were evaluated for the without stimulation condition only. When more than one cluster was present, we performed a Wilcoxon signed-rank test between pairs of clusters within study participants. All cluster combinations within a participant were tested for participants with at least two clusters (18 degrees of freedom).

### Phase-amplitude profiles

Phase-amplitude profiles summarise the change in tremor severity at each stimulation phase. These were derived solely from the accelerometer signals. To this end, we first computed the tremor envelope using the absolute value of the Hilbert transform of the band-pass filtered accelerometer signals. The change in tremor severity was derived from each 5-s stimulation segment by taking the difference between the average tremor envelope during the last 1 s of stimulation (i.e., 4–5 s) and that during the 1 s prior to stimulation onset (i.e., − 1 to 0 s), divided by the average tremor envelope during the 1 s prior to stimulation onset (Fig. 2). As such, − 1 indicates complete tremor suppression, 0 indicates no change in tremor and positive values indicate amplification of tremor. The median change in tremor severity for each of the 12 phases was then computed, creating phase-amplitude profiles. It should be noted that this procedure was repeated for each accelerometer axis, TOP (i.e., biomechanically defined cluster), and study participant.

We determined whether stimulation delivered at a certain phase of limb acceleration significantly modulated tremor by comparing the change in tremor severity during peripheral nerve stimulation to spontaneous changes in tremor severity during the without stimulation condition. Each phase-amplitude profile (calculated separately for different accelerometer axes and TOPs) was compared to the corresponding surrogate distribution (Additional file 1). We drew *n* random instances from the TOP-specific surrogate distribution where *n* was the average number of trials across the 12 phases, took the median across the *n* points, and repeated this process 1,000,000 times, creating a distribution with 1,000,000 points. We applied Bonferroni correction for 12 comparisons, thus determining whether stimulation at a certain phase significantly modulated tremor (Additional file 1).

To explore the relationship between phase-amplitude profiles at the group level, we computed the average Fisher-transformed correlation between (1) phase-amplitude profiles from different axes in the same cluster (for example, between axes *x* and *y* in cluster 1), and (2) between the same axes across clusters (for example, between axis *x* in cluster 1 and axis *x* in cluster 2).

### Re-aligned phase-amplitude profiles

The median change in tremor severity at each phase bin was classified as suppression when smaller than zero, and as amplification when greater than zero. Each phase amplitude profile (calculated separately for different accelerometer axes and TOPs) was realigned by mapping the point of minimum suppression or maximum amplification to 180°. Subsequently, the number of instances of suppression or amplification at each phase was summed across phase-amplitude profiles. This was normalised to a number between 0 and 1 by dividing the number of occurrences at each phase by that at 180°.

We generated surrogate phase-amplitude profiles for each accelerometer axis and TOP. To this end, we repeated the process used for generating the distribution with 1,000,000 points (please see ‘Phase-amplitude profiles’ in ‘materials and methods’ and Additional file 1) and then drew 12 points 1000 times from this distribution. This created 1000 surrogate phase-amplitude profiles (1000 by 12) for each participant, accelerometer axis and TOP. Every surrogate phase-amplitude profile (1 by 12) was realigned to the maximum or minimum change in tremor. This was achieved by mapping minimum suppression or maximum amplification to 180°. As before, we classified each bin as suppressive when the median change in tremor severity was smaller than zero and amplifying when greater than zero. We then summed the number of instances with suppression or amplification across the 1000 surrogate phase-amplitude profiles. We divided this sum by 1000, normalising it to a value between zero and one reflecting the probability of seeing suppression or amplification at that phase. These probabilities were compared, with a two-sample t-test, to those derived from data recorded during peripheral nerve stimulation and corrected for multiple comparisons using the false discovery rate (FDR) procedure.

## Results

### Tremor oscillation patterns

**Fig. 3:**
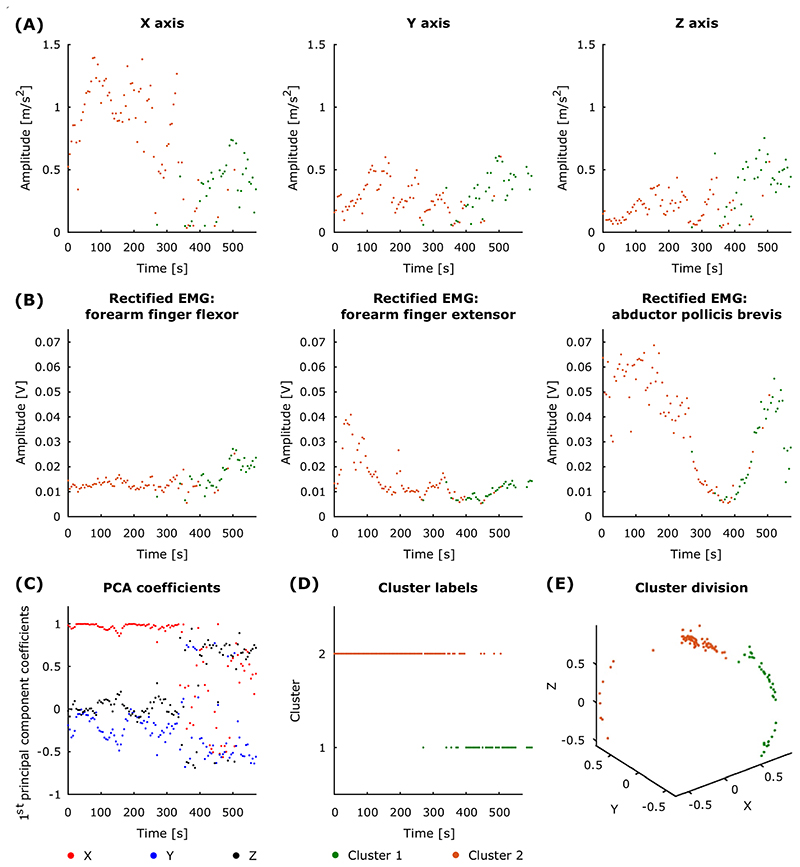
An example of how cluster representation in serial 5-s periods changes over time in the signals without stimulation. **A** Dominant cluster label (orange or green) obtained from the first principal component coefficients is shown for the median accelerometer signal amplitude and **B** for the median rectified EMG in each channel per 5-s period. **C** The first principal component coefficients corresponding to the three accelerometer axes. **D** The labels for the time segments assigned to each of the two clusters. **E** The corresponding three-dimensional cluster division from the first principal component coefficients

We first aimed to identify switches in tremor oscillation patterns (TOPs) such as a switch from a predominant tremor in the *x* dimension (i.e., pronation-supination) to one in the *z* dimension (i.e., extension-flexion). In seven out of 10 participants, the peripheral manifestation of tremor varied during the recording session and as a result we observed more than one TOP, indicated by the presence of more than one cluster delineating coefficients contributing to principal components of accelerometer recordings. Implicit in this result is that the dominant cluster (or clusters where the second principal component was also considered) changed over time in these participants exhibiting more than one cluster (Additional file 1). Figure 3 provides an example of how cluster representation in serial 5-s periods changes over time and includes the cluster label, the median tremor signal amplitude and the median rectified EMG envelope in each axis per 5-s period.

### Differences between tremor oscillation patterns

We next explored whether there were any physiological differences between clusters (i.e., TOPs) based on features of accelerometer and EMG recordings. Significant differences in tremor peak frequency and amplitude envelope were found for all three accelerometer axes (p ≤ 0.0003 for all three axes, as given by the Wilcoxon signed-rank test applied to the absolute value of the difference in tremor amplitude and peak frequency across clusters). EMG peak frequency and amplitude envelope also showed significant differences between clusters (p ≤ 0.0003 for all three muscles, as given by the Wilcoxon signed-rank test applied to the absolute value of the difference in EMG amplitude and peak frequency across clusters for the three muscles—abductor pollicis brevis, forearm finger flexors, and forearm finger extensors). It should be noted that the average duration of a cluster during the “without stimulation” condition was 100.5 ± 143.5 s (mean ± SD).

### Phase-specific response to peripheral stimulation

**Fig. 4:**
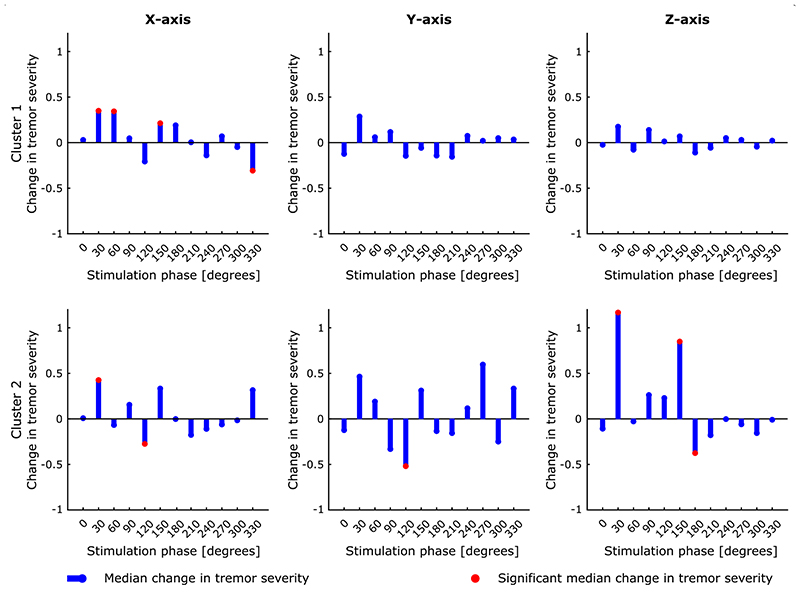
Example phase-amplitude profiles for one study participant, showing the median change in tremor severity at each phase for the three accelerometer axes and two clusters. Significant median change in tremor severity was identified with respect to the tremor variability during the without stimulation condition following Bonferroni correction for 12 comparisons

**Fig. 5:**
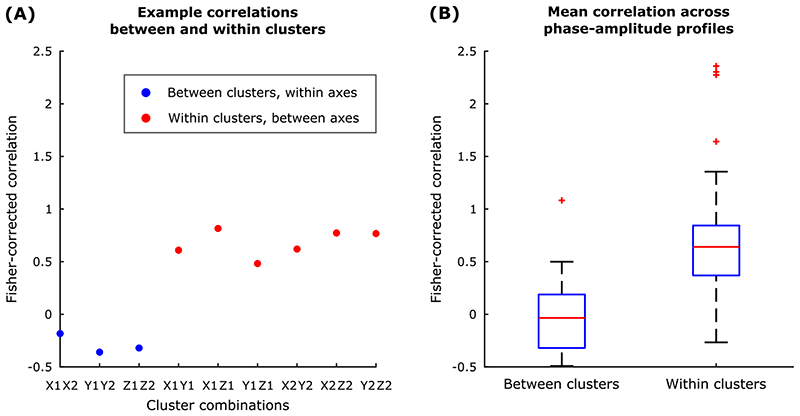
**A** Example of the Fisher-corrected correlations for each cluster combination in one study participant. **B** Mean between and within clusters of the Fisher-corrected correlation across participants

Having confirmed that TOPs differ in frequency and amplitude, we next explored how phase-locked stimulation modulated tremor severity. Figure 4 shows an example phase-amplitude profile derived for one participant. Stimulation significantly enhanced or reduced the instantaneous tremor severity depending on the stimulation timing. As highlighted by this example, the most effective stimulation phase, indicated by the phase which induces changes in tremor amplitude beyond natural variability of tremor, was more consistent across different axes of the same cluster (TOP) than across clusters (i.e., cluster 1 suppressive phase of 330° vs. cluster 2 suppressive phase of 120°). At the group level, the correlation between axes in the same cluster was greater than that across clusters (Fig. 5). The difference between the correlations within and between clusters was significant (p-value < 10^–7^ for a two-sample t-test). Thus although the pattern of modulation differs between clusters from the same hand, within a cluster the pattern of modulation is relatively conserved across axes, providing additional evidence that clusters are meaningful in terms of a common representation.

At the group level, significant tremor suppression occurred across 12 bins in 12 phase-amplitude profiles for five out of 10 participants, and significant amplification across 16 bins in 12 plots from six participants. In total, 28 bins (1.7%) displayed significant change in tremor severity (Table 2). This number is eight times above the chance level for Bonferroni correction, which was used to determine the significance of different bins in the phase-amplitude profiles. On average, phase-locked median nerve stimulation was able to significantly amplify tremor by 117% ± 243% (mean ± SD) and suppress it by 36% ± 9% (mean ± SD).

### Angle of stimulation

**Fig. 6:**
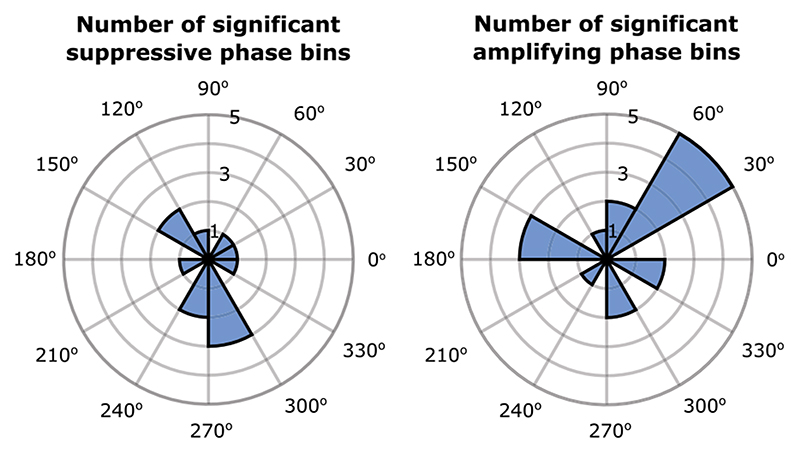
Rose plots indicating the number of phase bins for which there was significant tremor suppression or significant tremor amplification at each of the 12 equally spaced stimulation phases. Rayleigh test for departure from circular uniformity resulted in p = 0.7204 for suppression and p = 0.2347 for amplification

There was no systematically preferred suppressive or amplifying phase across study participants. Figure 6 shows the number of phase bins across study participants and clusters for which there was significant tremor suppression or significant tremor amplification, at each of the 12 equally spaced stimulation phases (Rayleigh test resulted in p = 0.7204 for suppression and p = 0.2347 for amplification).

**Fig. 7:**
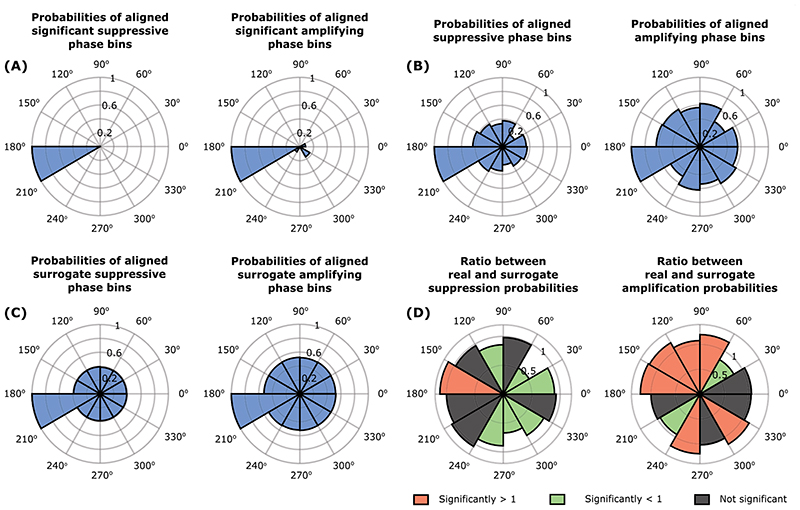
A Rose plots indicating the probability of significant tremor suppression or tremor amplification at each of the 12 equally spaced stimulation phases where the minimum suppression and maximum amplification angles were realigned to 180°. Rayleigh test resulted in p = 2e-8 for suppression and p = 0.0002 for amplification. B Rose plots indicating the probability of tremor suppression or tremor amplification at each of the 12 equally spaced stimulation phases, regardless of whether tremor modulation was significant, where the minimum suppression and maximum amplification angles in all phase-amplitude profiles were realigned to 180°. Rayleigh test resulted in p = 6e-6 for suppression and p = 0.0334 for amplification. C Rose plots generated from surrogate phase-amplitude profiles indicating the probability of tremor suppression or tremor amplification at each of the 12 equally spaced stimulation phases where the minimum suppression and maximum amplification angles were realigned to 180° regardless of whether tremor modulation were significant. D Rose plots displaying the ratio between the real (B) and surrogate (C) probabilities of suppression or amplification in all phase-amplitude profiles, such that ratios significantly greater than 1 after FDR correction are shown in red, ratios significantly smaller than 1 are shown in green, and ratios that are not significant after FDR correction are shown in grey. Precise p-values given in Table 3

Next, we considered re-aligned stimulation angles. As to be expected, separately aligning stimulation angles to the most suppressive or amplifying stimulation angle observed in each phase-amplitude profile produced non-uniform distributions at the group level (Fig. 7A and B). However, stimulation and its surrogate data (Fig. 7C) aligned to minima differed significantly, as did stimulation and surrogate data aligned to maxima (Fig. 7D). In the case of suppression, the 150–180° bins were significantly more likely to show amplitude reduction during stimulation than in surrogates. Conversely, the 0–60, 90–120, and 240–330° bins were significantly less likely to be associated with amplitude reduction during stimulation than with surrogates (Fig. 7D; Table 3). The overall pattern was less distinct in the case of amplification, although the probability of amplitude increases and decreases during stimulation was significant in the 60–180, 240–270, and 300–330° bins, respectively. It should be noted that for both suppression and amplification, these groupings are placed approximately 180° apart.

## Discussion

We have provided evidence that Parkinsonian rest tremor of the hand often exhibits distinct oscillatory patterns, and that the output of a given tremor oscillator can be modulated in amplitude by peripheral stimulation at specific phases, although these critical phases differ between tremor oscillators.

### Multiple tremor oscillators

The existence of independent oscillators driving different peripheral tremor components was inferred from the pattern of tremulous wrist movement in space which could be divided into more than one pattern by principal component and cluster analysis in seven out of ten study participants. The independent nature of these patterns or clusters was supported by cluster separation, significant differences in frequency distribution, significant differences in associated muscle activations, and through differences in the phase-amplitude profiles observed during phase-locked peripheral nerve stimulation. Moreover, the mean correlations across phase-amplitude profiles indicate that the amplitude modulation patterns were more similar across different axes in the same cluster than across the same axis from different clusters. Clusters were dynamic with their likelihood of being dominant altering over time. The latter is in keeping with the inconstancy of coherence between neurons oscillating at tremor frequency, and the inconstancy of the coherence between these neurons and background activity in the STN [47].

Although Parkinsonian rest tremor is considered to represent the effect of independent oscillators in different limbs [27–31], the current study suggests that central tremor oscillators could potentially adopt a much finer structure so that multiple oscillators may contribute to the tremor in a given hand. Electroencephalography and/or local field potentials should be evaluated in order to establish the link between central tremor oscillators and the changes in the pattern of tremulous wrist movement.

Could the presence of more than one cluster during stimulation be due to contamination of the tremulous wrist movements in space by a direct response to stimulation? This is unlikely as we only accepted clusters that were present both with and without peripheral nerve stimulation. In addition, significant differences in frequency and amplitude were found between clusters in the cluster division obtained from the recordings without stimulation. Furthermore, stimulation was performed at just below the motor threshold, so that direct responses were small and inconstant.

### Contextual factors shape tremor dynamics

One paradoxical observation is that there was no systematic phase at which peripheral stimulation induced tremor amplification or suppression across study participants. Hence significant effects were only evident after phase-amplitude profiles were realigned to their maxima or minima. This was also the case with stimulation of the ventrolateral thalamus in people with essential tremor [39]. It has previously been shown that median nerve stimulation can induce spiking activity in parts of the thalamus which are also implicated in tremor in Parkinson’s disease [21–25]. Therefore, the precise effect of median nerve stimulation could be influenced by the conduction delays between the spindle afferents and the thalamus. Since stimulation is phase-locked to limb acceleration, the precise relationship between tremor and thalamic tremor cells could also impact stimulation effects [23, 26]. These sources of variation across participants could all contribute to the inconsistencies in stimulation phases that significantly modulated tremor (Fig. 6 and Additional file 1). As demonstrated here, effective stimulation parameters such as the stimulation phase should therefore be determined individually to mitigate this variability across participants. An open question is whether or not systematic phases, those at which stimulation induces tremor amplification or suppression, will emerge if we quantify effects from EMG rather than accelerometer signals. However, this is not trivial as it requires the identification and recording of EMG signals that are relatively selectively involved in one or other cluster.

We should also highlight two important assumptions made in our analysis. The first is that there are no more than two clusters each in the first or second principal components underlying rest tremor of the hand, although this assumption was supported by the distance between the clusters. Second, we assume that the activity of one cluster in each principal component dominates during each 5-s epoch of analysis. Although additional clusters cannot be excluded, their impact is likely to be small. However, we have little evidence to assume that a given cluster consistently dominates throughout each of the arbitrarily defined 5-s epochs of analysis. The presence of one or more additional active clusters within an epoch may again influence the phase at which any amplification or suppression effect predominates, and this confound will not necessarily be addressed by considering EMG rather than accelerometer activity.

### Phase-dependent modulation of tremor

At the individual level phase-amplitude profiles contained more bins with significant tremor amplitude amplification or suppression than could be accounted for by chance. Realignment of effects to the phase bin that afforded maximum amplitude reduction or increase in phase-amplitude profiles highlighted two groupings of phases, associated with significant decreases in the probability of suppression compared to surrogates and placed approximately 180° apart. A comparable effect was evident with regard to the probability of stimulation-induced tremor amplification. These observations raise the possibility that each TOP (or cluster) is underpinned by an oscillator with a frequency twice that of the limb tremor, and that peripheral stimulation is interacting with this harmonic. Such a harmonic could be centrally represented in line with the pattern of tremor modulation reported with phase-locked transcranial alternating current stimulation in people with Parkinsonian rest tremor [6]. Direct recordings of brain activity also point to a coupling of peripheral tremor to central oscillatory activity at twice tremor frequency [48–50].

The present results suggest that provided the dominant tremor oscillation is tracked in time, then phase-locked peripheral nerve stimulation may be able to attenuate tremor amplitude. For those study participants with one TOP, determining the stimulation phase to suppress tremor and continuously delivering stimulation phase-locked to this instance would be straightforward. However, the remaining participants in our cohort had two or more tremor components, the dominance of which fluctuated over time. Accordingly, successful stimulation would have to dynamically match the phase to maximally attenuate tremor to the tremor component dominating at that moment in time, a relationship which could perhaps be established through machine learning. But that is not the only issue impeding the development of phase-locked stimulation at the wrist as a non-invasive therapeutic option. The degree of tremor attenuation achieved by phase-locked stimulation when this was maintained at the appropriate phase for 5-s periods is also modest (36% suppression). Sustaining stimulation at the optimal phase for longer may potentially improve the degree of attenuation [6, 39], as might searching for the optimal phase for attenuation with finer phase resolution and determining changes in tremor clusters with finer temporal resolution. Finally, given that coupling of peripheral tremor to central oscillatory activity may be at twice tremor frequency, then trials of stimulation that is phase-locked to this central harmonic are also warranted. This would require the determination of sensitive phases from phase-amplitude profiles derived by stimulating at twice the frequency of TOPs in the periphery.

### Limitations of the current study

We have already alluded to the importance of polymyography when investigating any consistency between stimulation phases promoting maximal suppression and amplification of tremor amplitude. Evaluation of changes in central tremor oscillators through electrocorticography and local field potentials will be needed in order to confirm the relationship between peripheral changes in tremor form and central rhythms. Another related limitation of our analyses is that temporal resolution was 5 s which may be too coarse to capture rapid shifting between tremor clusters and is likely to only reveal which TOP dominates within each temporal window. This might also explain the complex form of many phase-amplitude profiles and why they have relatively wide confidence limits as the 5-s analysis windows may be capturing more than one tremor component. Another limitation of our study is that electrical stimulation might have caused sub-motor threshold activation of wrist nerves (i.e., the radial and ulnar nerves) other than the intended target (i.e., the median nerve). Whereas any unforeseen consequences of this activation were minimised by keeping the stimulation below the motor threshold, it would be valuable to characterise the effects of stimulating the median vs. radial or ulnar nerves by intentionally targeting these nerves. In addition, our cohort was relatively small, and we did not investigate the effects of movement or posture on TOPs. It therefore remains to be seen whether these latter conditions change the precise distribution of spinal motor neurons recruited within a cluster, and thereby modify the tremor trajectory in space. Finally, our small cohort does not allow us to explore any relationship between disease progression or phenotype, and the number and organisation of tremor clusters.

### Challenges and future directions

Tremor shows great variability within and across individuals. Peripheral stimulation parameters that effectively modulate tremor may therefore vary across people with Parkinson’s disease and resting tremor, but critically may also vary in time as demonstrated in this study. In order to maximise peripheral stimulation efficacy, stimulation parameters should be optimised individually. Similarly, to account for potential changes in tremor form, which could influence efficacy of peripheral stimulation, parameters for this non-invasive therapy may need to be occasionally adjusted. Considering the vast number of parameter combinations (e.g., pulse width, amplitude, frequency and pattern), peripheral stimulation parameters may need to be optimised outside of the laboratory setting using a wearable peripheral stimulator that is capable of remote parameter optimisation. Such an adaptive system could also be used to track an individual’s tremor form in real time using percentage tremor power in different accelerometer axes and adjust stimulation parameters accordingly in order to sustain stimulation efficacy. Automated parameter optimisation schemes such as Bayesian parameter optimisation could be critical in this context in order to achieve parameter optimisation both across individuals and time [51].

## Conclusions

We have furnished evidence that Parkinsonian hand tremor may be the product of multiple oscillators leading to discrete patterns of acceleration in three-dimensional space. Oscillators tend to be sensitive to peripheral stimulation, and this leads to discrete patterns of stimulationphase dependent suppression and amplification. These observations are important in helping to explain tremor variability, and need to be taken into account if phase-locked stimulation is to be developed as a potential therapeutic intervention to suppress Parkinsonian resting tremor [6, 39]. In particular, the suppressive phase of stimulation may change according to which oscillator dominates at a particular moment in time. The issue of multiple oscillators may be relevant to the development of treatments in other neurological disorders such essential and dystonic tremor, where phase-locked stimulation has been explored as a potential therapeutic technique (15, 16, 39, 40).

## Abbreviations

AD: Analogue-to-digital
EMG: Electromyography
FDR: False discovery rate
PCA: Principal component analysis
STN: Subthalamic nucleus
TOP: Tremor oscillation pattern.

## Supplementary Material

Additional File 1

## Figures and Tables

**Table 1:** Individual study participant’s characteristics

Participant	1	2	3	4	5	6	7	8	9	10	Mean
Age [years]	75	64	55	56	75	63	62	75	57	73	65.5
Gender	Female	Female	Male	Male	Male	Female	Male	Female	Female	Female	-
Arm tested	Left	Right	Right	Right	Right	Right	Right	Left	Right	Right	-
Parkinson’s disease medication state	On	On, skipped last dose	Off	On, skipped last dose	Off	On, skipped last dose	On, skipped last dose	On	On, skipped last dose	On	-
Likert scale assessment	Strongly agree	Strongly agree	Agree	Strongly agree	Strongly agree	Strongly agree	Strongly agree	Strongly agree	Strongly agree	Strongly agree	-
Tremor amplitude (mean±SD) [m/s^2^]	3.9 ±2.9	6.2 ±2.4	3.0 ±2.2	15.9 ±3.2	0.7 ±0.4	4.7 ± 1.6	0.4 ±0.2	2.0 ±0.6	7.3 ±2.7	8.1 ±3.1	5.2
Peak tremor frequency [Hz]	5	4	5	5	5	5	5	5	5	6	5

Note stimulation sensation was assessed using the following Likert scale: “The stimulation was easily tolerated: (A) Strongly disagree; (B) Disagree; (C) Neither agree nor disagree; (D) Agree; (E) Strongly agree”

**Table 2:** Summary of number of phase bins, plots and study participants displaying significant tremor suppression or amplification, as well as the number of bins and plots expected at the chance level considering that the Bonferroni correction was applied

	Significant suppression	Significant amplification	Chance level
Bins	12/828	16/828	3.45/1656
Plots	12/69	12/69	3.45/138
Participants	5/10	6/10	1/20

**Table 3:** Significance values corresponding to Fig. 7 where rose plots display the ratio between the real (Fig. 7B) and surrogate (Fig. 7C) probabilities of all phase bins

Phase	Suppression	Amplification
0	0.0071*	0.0425
30	0.0000*	0.0000*
60	0.5763	0.0000*
90	0.0001*	0.0055*
120	0.3972	0.0000*
150	0.0022*	0.0000*
180	1	1
210	0.1875	0.0273*
240	0.0045*	0.0000*
270	0.0000*	0.4148
300	0.0000*	0.0000*
330	0.0522	0.0836

Significance values were obtained from paired-sample t-tests between the probability for suppression or amplification for each study participant, axis, and cluster at each phase of the surrogate data, and the probability of suppression or amplification across all participants, axes and clusters for each phase of the real aligned data

* P-values that remain significant after correction by the FDR procedure

## Data Availability

The datasets generated and/or analysed during the current study are available from the corresponding author on reasonable request.
