# Supplementary material for: Identifying and modulating distinct tremor states through peripheral nerve stimulation in Parkinsonian rest tremor
Source: J Neuroeng Rehabil. Author manuscript; Available in PMC 2022 Jan 11. (PMC8709974; doi:10.1186/s12984-021-00973-6)
Supplement: Additional File 1 [file EMS140701-supplement-Additional_File_1.docx]

**Additional Information**

**Additional Methods:**

*Tremor Oscillation Patterns*

In order to determine tremor oscillation patterns (TOPs), the triaxial accelerometer signals from both without stimulation and during peripheral nerve stimulation conditions were divided into 5-second segments. Principal component analysis (PCA) was applied to each 5-second segment. For the peripheral nerve stimulation condition, 5-second segments corresponded to instances when stimulation was delivered at one of the 12 specific phases. For the without stimulation condition, 5-second segments corresponded to consecutive time segments without overlap (*Additional Figure 1*).

*Tremor Oscillation Patterns and Phase-Amplitude Profiles*

In order to determine whether stimulation delivered at a certain phase of limb acceleration significantly modulated tremor, we compared the change in tremor severity during peripheral nerve stimulation to spontaneous changes in tremor severity during the without stimulation condition. To this end, 50,000 5-second segments were drawn from the without stimulation condition. These segments were used to create a surrogate distribution. As before, the change in tremor severity was defined as the difference between the average tremor severity during the last 1 second of the segment (i.e., 4 to 5 seconds) and that during the 1 second prior to segment onset (i.e., -1 to 0 seconds), divided by the average tremor severity during the 1 second prior to segment onset. It should be noted that some of these segments could partially overlap in time (*Additional Figure 1*).

In order to determine the interaction between TOPs and phase-amplitude profiles, 5-second segments from the without stimulation condition, which were used to form the surrogate distribution, were concatenated with 5-second segments from the peripheral nerve stimulation condition and subjected to cluster analysis as outlined in the *Materials and Methods*, allowing us to split the surrogate distribution according to TOPs and to compare phase-amplitude profiles from each TOP to a surrogate distribution derived from the same cluster.

**Pipeline to generate the phase-amplitude profiles, their confidence limits, and the statistical comparisons between clusters**

**
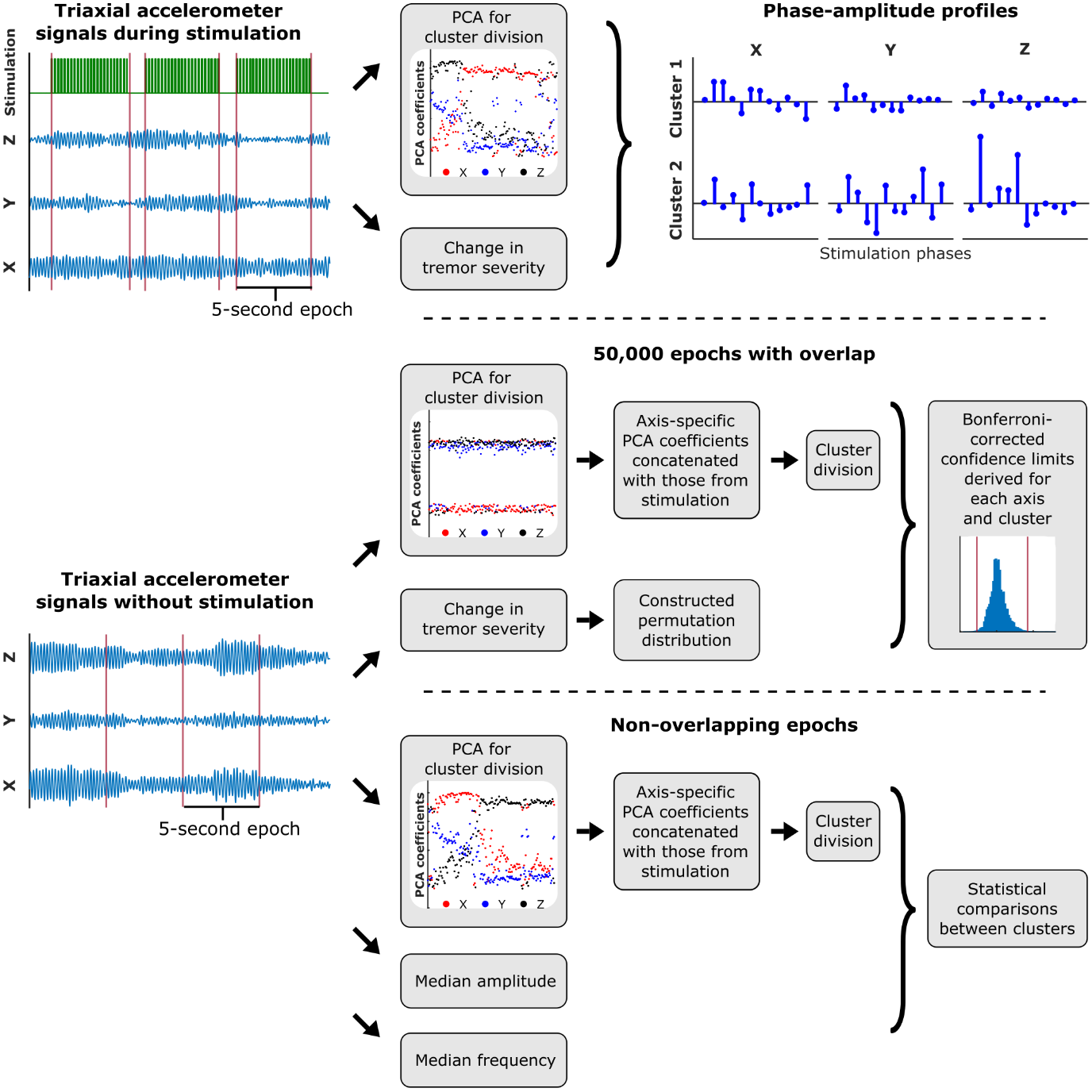
**

*Additional Figure 1: Summary of the analysis pipeline to generate the phase-amplitude profiles, their confidence limits, and the statistical comparisons between clusters.*

**Additional Results:**

TOPs reflected contributions (i.e. coefficients) of each tremor axis to the first principal component. The median percentage of the total variance that was explained by the first principal component was 91.6 ± 4.8% (mean ± SD) across 5-second epochs derived from the without stimulation condition. In six out of 10 participants, the first principal component accounted for a median of at least 90% of the total variance.

By also taking the second principal component in the remaining four participants, we ensured that over 90% of the total variance of the tremor was captured in every participant. In these four participants, the second component explained 12.2 ± 1.9% (mean ± SD) of the total variance.

Eliminating any clusters which contained less than 10% of the total number of segments obtained during the peripheral nerve stimulation condition, five out of 10 participants exhibited two clusters in the first principal component. Four participants exhibited two additional clusters in the second principal component resulting in 23 clusters in total. These clusters were well separated, with a mean Silhouette score of 0.80 ± 0.16 (mean ± SD), and the optimal number of clusters being equal to two for each considered principal component.

**Phase bins in which significant suppression occurred for each phase-amplitude profile**

| **1^st^ principal component** | | | | | | **2^nd^ principal component** | | | | | |  |
| --- | --- | --- | --- | --- | --- | --- | --- | --- | --- | --- | --- | --- |
| **Cluster 1** | | | **Cluster 2** | | | **Cluster 1** | | | **Cluster 2** | | |  |
| **Participant** | X | Y | Z | X | Y | Z | X | Y | Z | X | Y | Z |
| 1 | - | - | - | * | | | - | - | - | - | - | - |
| 2 | * | | | 270 | 240 | 240 | * | | | * | | |
| 3 | - | - | - | - | - | - | * | | | * | | |
| 4 | * | | | - | - | - | * | | | * | | |
| 5 | - | - | - | - | - | - | - | - | - | - | - | - |
| 6 | - | - | - | * | | | * | | | * | | |
| 7 | * | | | - | - | 30 | - | - | - | - | - | - |
| 8 | 330 | - | - | 120 | 120 | 180 | * | | | * | | |
| 9 | - | - | - | - | - | 90 | * | | | * | | |
| 10 | - | - | - | - | - | 270 | 0 | - | - | - | - | 270 |

*Additional Table 1: Phase bins (in degrees) in which there was significant tremor suppression for each phase amplitude profile – corresponding to each study participant, principal component, cluster, and tremor axis. Note that (*) indicates that the corresponding cluster or principal component was not considered in the analysis for that study participant, and (-) indicates that the corresponding cluster or principal component was considered for the analysis, but there was no significant tremor suppression for that phase amplitude profile.*

**Phase bins in which significant amplification occurred for each phase-amplitude profile**

| **1^st^ principal component** | | | | | | **2^nd^ principal component** | | | | | |  |
| --- | --- | --- | --- | --- | --- | --- | --- | --- | --- | --- | --- | --- |
| **Cluster 1** | | | **Cluster 2** | | | **Cluster 1** | | | **Cluster 2** | | |  |
| **Participant** | X | Y | Z | X | Y | Z | X | Y | Z | X | Y | Z |
| 1 | - | - | - | * | | | - | - | - | - | - | 30 |
| 2 | * | | | - | - | - | * | | | * | | |
| 3 | - | - | - | - | - | - | * | | | * | | |
| 4 | * | | | - | - | - | * | | | * | | |
| 5 | - | - | - | - | - | - | - | - | - | - | - | - |
| 6 | - | - | 150 | * | | | * | | | * | | |
| 7 | * | | | 270 | 90 270 | 330 | 330 | - | - | - | - | - |
| 8 | 30  60  120 | - | - | 30 | - | 30  150 | * | | | * | | |
| 9 | 30 | - | - | - | - | 210 | * | | | * | | |
| 10 | - | - | - | - | - | - | - | 60 | - | - | - | - |

*Additional Table 2: Phase bins (in degrees) in which there was significant tremor amplification for each phase amplitude profile – corresponding to each study participant, principal component, cluster, and tremor axis. Note that (*) indicates that the corresponding cluster or principal component was not considered in the analysis for that study participant, and (-) indicates that the corresponding cluster or principal component was considered for the analysis, but there was no significant tremor amplification for that phase amplitude profile.*
